# Immune signatures and disorder-specific patterns in a cross-disorder gene expression analysis

**DOI:** 10.1192/bjp.bp.115.175471

**Published:** 2016-09

**Authors:** Simone de Jong, Stephen J. Newhouse, Hamel Patel, Sanghyuck Lee, David Dempster, Charles Curtis, Jose Paya-Cano, Declan Murphy, C. Ellie Wilson, Jamie Horder, M. Andreina Mendez, Philip Asherson, Margarita Rivera, Helen Costello, Stefanos Maltezos, Susannah Whitwell, Mark Pitts, Charlotte Tye, Karen L. Ashwood, Patrick Bolton, Sarah Curran, Peter McGuffin, Richard Dobson, Gerome Breen

**Affiliations:** **Simone de Jong**, PhD, **Stephen J. Newhouse**, PhD, **Hamel Patel**, **Sanghyuck Lee**, **David Dempster**, **Charles Curtis**, MSc, **Jose Paya-Cano**, PhD, MRC Social, Genetic & Developmental Psychiatry Centre, Institute of Psychiatry, Psychology & Neuroscience, King's College London and NIHR Biomedical Research Centre for Mental Health, Maudsley Hospital and Institute of Psychiatry, Psychology & Neuroscience, King's College London, UK; **Declan Murphy**, MD, The Sackler Institute for Translational Neurodevelopment, Department of Forensic and Neurodevelopmental Sciences, Institute of Psychiatry, Psychology & Neuroscience, King's College London, UK; **C. Ellie Wilson**, PhD, The Sackler Institute for Translational Neurodevelopment, Department of Forensic and Neurodevelopmental Sciences, Institute of Psychiatry, Psychology & Neuroscience, King's College London UK and Individual Differences, Language and Cognition Lab, Department of Developmental and Educational Psychology, University of Seville, Spain; **Jamie Horder**, PhD, **M. Andreina Mendez**, PhD, The Sackler Institute for Translational Neurodevelopment, Department of Forensic and Neurodevelopmental Sciences, Institute of Psychiatry, Psychology & Neuroscience, King's College London; **Philip Asherson**, PhD, MD, MRC Social, Genetic & Developmental Psychiatry Centre, Institute of Psychiatry, Psychology & Neuroscience, King's College London, UK; **Margarita Rivera**, PhD, MRC Social, Genetic & Developmental Psychiatry Centre, Institute of Psychiatry, Psychology & Neuroscience, King's College London, UK and CIBERSAM-University of Granada and Instituto de Investigación Biosanitaria ibs. GRANADA. Hospitales Universitarios de Granada/Universidad de Granada, Granada, Spain; **Helen Costello**, PhD, Wolfson Centre for Age Related Diseases, Institute of Psychiatry, Psychology & Neuroscience, King's College London, UK; **Stefanos Maltezos**, MSc, MD, **Susannah Whitwell**, MD, **Mark Pitts**, Adult ADHD Service, South London and Maudsley NHS Foundation Trust, London, UK; **Charlotte Tye**, PhD, Department of Child & Adolescent Psychiatry, Institute of Psychiatry, Psychology & Neuroscience, King's College London, UK; **Karen L. Ashwood**, PhD, Brighton and Sussex Medical School, University of Sussex, Brigton, UK; **Patrick Bolton**, PhD, MD, Department of Child & Adolescent Psychiatry, Institute of Psychiatry, Psychology & Neuroscience, King's College London, UK; **Sarah Curran**, PhD, MD, Department of Child & Adolescent Psychiatry, Institute of Psychiatry, Psychology & Neuroscience, King's College London and Brighton and Sussex Medical School, University of Sussex, Brighton, UK; **Peter McGuffin**, PhD, MD, MRC Social, Genetic & Developmental Psychiatry Centre, Institute of Psychiatry, Psychology & Neuroscience, King's College London, UK; **Richard Dobson**, PhD, **Gerome Breen**, PhD, MRC Social, Genetic & Developmental Psychiatry Centre, Institute of Psychiatry, Psychology & Neuroscience, King's College London and NIHR Biomedical Research Centre for Mental Health, Maudsley Hospital and Institute of Psychiatry, Psychology & Neuroscience, King's College London, UK

## Abstract

**Background**

Recent studies point to overlap between neuropsychiatric disorders in symptomatology and genetic aetiology.

**Aims**

To systematically investigate genomics overlap between childhood and adult attention-deficit hyperactivity disorder (ADHD), autism spectrum disorder (ASD) and major depressive disorder (MDD).

**Method**

Analysis of whole-genome blood gene expression and genetic risk scores of 318 individuals. Participants included individuals affected with adult ADHD (*n* = 93), childhood ADHD (*n* = 17), MDD (*n* = 63), ASD (*n* = 51), childhood dual diagnosis of ADHD–ASD (*n* = 16) and healthy controls (*n* = 78).

**Results**

Weighted gene co-expression analysis results reveal disorder-specific signatures for childhood ADHD and MDD, and also highlight two immune-related gene co-expression modules correlating inversely with MDD and adult ADHD disease status. We find no significant relationship between polygenic risk scores and gene expression signatures.

**Conclusions**

Our results reveal disorder overlap and specificity at the genetic and gene expression level. They suggest new pathways contributing to distinct pathophysiology in psychiatric disorders and shed light on potential shared genomic risk factors.

Psychiatric disorders are leading causes of disability globally, surpassing that attributed to more physical disorders. Worldwide prevalence rates for the five major psychiatric disorders range from 0.2% for attention-deficit hyperactivity disorder (ADHD) to 5.7% for major depressive disorder (MDD), with schizophrenia, autism spectrum disorder (ASD) and bipolar disorder showing intermediate prevalence.^1^ Although these disorders are classified into distinct disorder categories, they show overlap in symptomatology and shared genetic risk in family studies, as well as comorbidity in individuals and families.^2^ For example, 41% of patients with ASD were found to have a comorbid disorder, including ADHD.^3^ In addition, family studies show that relatives of probands with bipolar disorder show an increased risk for both bipolar disorder and schizophrenia.^4^ This could indicate common aetiological pathways for some psychiatric disorders and studying them jointly could lead to new insights.

Twin study heritability estimates for psychiatric disorders are moderate for MDD (~40%) and high (80–90%) for ADHD, ASD and schizophrenia, indicating a significant genetic component in their aetiology.^5–7^ Large genome-wide studies have now started to reveal risk variants for individual disorders but an outstanding question is whether genetic risk factors or polygenic risk scores are specific to particular disorders, or shared. For instance, certain copy number variations (CNVs) are overrepresented in both patients with schizophrenia and those with ASD.^8,9^ Additionally, calcium-channel activity genes have been shown to have shared involvement in ASD, schizophrenia and bipolar disorder, whereas polygenic risk analysis showed strong overlap of genome-wide risk between bipolar disorder, MDD and schizophrenia, and moderate overlap between ASD and schizophrenia.^10^ In another sample, high sharing was seen between bipolar disorder and schizophrenia, with moderate overlap between MDD and schizophrenia, bipolar disorder and ADHD and low, but significant, overlap between schizophrenia and ASD.^11^ These results suggest that some shared molecular genetic factors underlie a significant proportion of the risk for development of several psychiatric disorders. To date, however, it is not known whether these genomic overlaps are reflected in overlapping patterns of gene expression in tissues. In this study we therefore explored whole blood gene expression across adult and childhood ADHD, ASD, MDD and healthy controls, using weighted gene co-expression analysis to search for patterns of correlated gene expression between disorders as well as disorder-specific gene expression signatures. In addition, we generate polygenic risk scores to assess overlap between disorders on a genome-wide genetic-risk level. We next investigate whether differences in polygenic risk scores are reflected in disorder-related gene expression profiles and whether they can be used to tease apart genetic and environmental influences on gene expression.

## Method

### Participants

Participants were from four different projects and included individuals affected with adult ADHD, childhood ADHD, MDD, ASD, childhood dual diagnosis ADHD–ASD and healthy controls (Table 1). Because of the age difference and its possible confounding effect on gene expression within the ADHD diagnostic group we decided to split the ADHD samples into a childhood (mean age 10 years) and adult (mean age 32 years) ADHD groups. Participant characteristics and inclusion/exclusion criteria used by each project are included in online supplement DS1. Briefly, we included: (a) 63 people with MDD and 57 controls from the Depression Case–Control (DeCC) study, a large case–control study that recruited unrelated patients from three clinical sites in the UK; (b) 93 adults with ADHD attending a National adult ADHD out-patient clinic in London, UK; (c) 16 individuals with ASD–ADHD, 7 with ASD, 17 with childhood ADHD and 7 controls from the Biomarkers for Childhood Neuropsychiatric Disorders (BioNed) project; and (d) 44 people with ASD and 14 controls from the Autism Interventions (AIMS) project. The following phenotypic information was available within each project and subsequently used for the current cross-disorder analyses: age, gender, diagnosis, date of collection, ethnicity, psychoactive medication use and, for all projects except DeCC, comorbidity of other psychiatric disorders (another psychiatric diagnosis was an exclusion criterion in the DeCC study). Each project has ethical approval and full informed consent for each participant (details in online supplement DS1). Whole blood samples were collected using PAXgene tubes for RNA and EDTA for DNA. All RNA samples were processed within one batch to generate whole-genome gene expression data.

**Table 1:** Sample characteristics

Diagnosis	*n*	% male	Age (s.d.)	RIN (s.d.)	% medication-free
Controls	78	59	46 (15)	8.3 (0.4)	97

Autism spectrum disorder	51	90	29 (11)	8.5 (0.3)	61

Major depressive disorder	63	33	47 (9)	8.5 (0.4)	29

Adult attention-deficit hyperactivity disorder	93	77	32 (12)	8.5 (0.5)	63

Childhood attention-deficit hyperactivity disorder	17	100	10 (2)	8.6 (0.4)	29

Autism spectrum disorder-attention-deficit hyperactivity disorder	16	100	11 (2)	8.7 (0.3)	69

RIN, RNA integrity number.

### Gene expression data preprocessing

Whole-genome gene expression data of a total of 424 individuals were generated using the Illumina HT-12.v4 BeadChips at the SGDP/BRC BioResource Illumina core lab according to the manufacturer's protocol. We rigorously quality controlled and preprocessed the data using a standard pipeline (https://github.com/snewhouse/BRC_MH_Bioinformatics), excluding sample and probe outliers, after which robust spline normalisation and log^2^ transformation were applied.^12^ After excluding samples based on low-quality expression profiles we included only participants with full phenotype data (age, gender, diagnosis, date of collection, ethnicity, psychoactive medication use per individual, and RNA integrity number (RIN) and RNA concentration per sample). This left 318 participants and 5638 probes for analysis (Table 1). To minimise project collection and sample handling batch effects, the data were corrected for three unknown variables captured by the SVA R package^13^ using a linear model. Surrogate variable (SV)1 was not correlated to known covariates, but SV2 and SV3 were highly correlated to cell proportion estimates by the CellMix package:^14^ SV2 (neutrophils *r* = −0.45, *P* = 4.5 × 0^−17^, monocytes *r* = 0.42, *P* = 1.2 × 10^−14^); SV3 (neutrophils *r* = −0.45, *P* = 4.4 × 10^−17^, lymphocytes *r* = 0.41, *P* = 2.4 × 10^−14^). No correlation of surrogate variables or principal components was found with self-reported ethnicity (*n* = 29, 9% of participants reported ethnicity other than White).

### Weighted gene co-expression network reconstruction

Corrected gene expression data were analysed using weighted gene co-expression analysis (WGCNA).^15,16^ We constructed a signed weighted co-expression network based on the matrix of pairwise Pearson correlation coefficients, which were raised to a fixed power (β = 12) by the criteria described by Zhang & Horvath.^16^ Soft-thresholding results in a 5638 × 5638 dimensional weighted adjacency matrix containing pairwise connection strengths. Subsequently, a topological overlap measure is calculated based on the number of shared neighbours. Modules were then defined as branches of a hierarchical clustering tree using a dissimilarity measure (1 – topological overlap). Each module is subsequently assigned a colour. To define a representative module expression profile for each module, we summarised the (standardised) gene expression profiles of the module by their first principal component. This statistic is referred to as the module eigengene: it can be thought of as an average gene expression value for all probes in a module per sample. In order to identify hub genes, we calculated a connectivity measure (‘module membership’) per probe by correlating expression values with the relevant module eigengene. Probes with high module membership are defined as ‘hub genes’ of their module.

### Module eigengenes and phenotypes of interest

The module eigengenes were correlated to phenotype of interest using a linear model.^17^ Participants were assigned a main diagnosis of a particular disorder (controls, ASD, MDD, adult ADHD, childhood ADHD, ADHD–ASD) *v.* all other participants. We investigated the effect of possible covariates on the association of module eigengenes with our phenotypes of interest. This led us to correct for gender, RIN and RNA concentration. Medication use defined as dichotomous measures of use of antidepressants (*n* = 73), stimulants (*n* = 49), antipsychotics (*n* = 7), benzodiazepines (*n* = 5) and mood stabilisers (*n* = 5) were investigated as a possible covariate. Considering the nature of the sample collection of the childhood ADHD–ASD and childhood ADHD samples, analyses were repeated also including age as covariate, allowing us to tease out diagnosis and age-specific effects. We used a Bonferroni threshold for significance (the number of tests was calculated as the number of modules × number of diagnoses × 2).

### Characterisation of modules of interest

Modules of interest were tested for enrichment of blood cell type lists using the userListEnrichment function in WGCNA package with five as minimum number of genes in a pathway. This function compares the number of overlapping genes to the maximal possible overlap and applies Bonferroni correction. Enrichment analyses were performed in WebGestalt using Wikipathways database.^18^ In addition, we performed connectivity mapping on selected modules to investigate overlap with drug-induced gene expression changes through the LINCS/CMap database (http://apps.lincscloud.org).^19^ We entered the gene content of modules of interest as upregulated, resulting in a list of compounds of which the application to cell lines results in a similar gene expression pattern.

### Genotype information and previous genome-wide association study (GWAS) findings

For most individuals genotype data were available and after quality control and imputation polygenic risk score analysis and genetic pathway analysis was carried out to investigate the relationship of genome-wide risk to disorder-specific gene expression findings in this study (see online supplement, Supplements DS2–4 and Figs DS1 and DS2).

## Results

The WGCNA on 318 participants and 5638 probes resulted in seven gene co-expression modules, ranging from 2077 probes in the turquoise module to 80 probes in the red module. The grey module contains 675 probes not belonging to any other module, representing background noise. The network dendrogram is given in online Fig. DS3 and all probes and corresponding module assignments in online Table DS1. The module eigengenes representing a summary of all genes in a given module were related to our traits of interest: participants with a main diagnosis of a particular disorder (controls, ASD, MDD, adult ADHD, childhood ADHD, ADHD–ASD) *v.* all other participants, covarying for gender, RIN, concentration in one model and also including age in a second model. This results in 7 phenotypes × 6 module eigengenes = 42 tests per model, therefore 84 tests in total when considering both models. We applied a Bonferroni threshold for significance of *P* = 0.05/84 = 6 × 10^−4^. Results for the first model are listed in Table 2. We did not find significant gene expression effects for the ASD and ADHD–ASD groups. In addition, medication use (antidepressants, stimulants, antipsychotics, mood stabilisers or benzodiazepines) was not significantly associated to any gene co-expression module (online Fig. DS4).

**Table 2:** Significant module trait associations^a^

		*t* (*P*)					
Module	Probes, *n*	Controls	Major depressive disorder	Adult attention-deficit hyperactivity disorder	Childhood attention-deficit hyperactivity disorder	Autism spectrum disorder–attention-deficit hyperactivity disorder	Autism spectrum disorder
Turquoise	2077	ns	4.4 (1.4 × 10^−5^)^b^	ns	4.0 (5.8 × 10^−5^)^b^	ns	ns

Red	80	ns	−4.3 (1.5 × 10^−5^)^c^	4.3 (2.2 × 10^−5^)^b^	ns	ns	ns

Green	186	ns	−5.8 (8.8 × 10^−9^) ^c^	4.8 (1.5 × 10^−6^)^b^	ns	ns	ns

Blue	1672	ns	ns	ns	−4.0 (6.7 × 10^−5^) ^c^	ns	ns

ns, not significant.

a. Module eigengenes were tested for association with phenotypes of interest including gender, RNA Integrity Number RIN and RNA concentration as covariates.

b. Positive associations.

c. Negative associations.

### The green and red immune modules are inversely correlated to psychiatric disorders

**Fig. 1:**
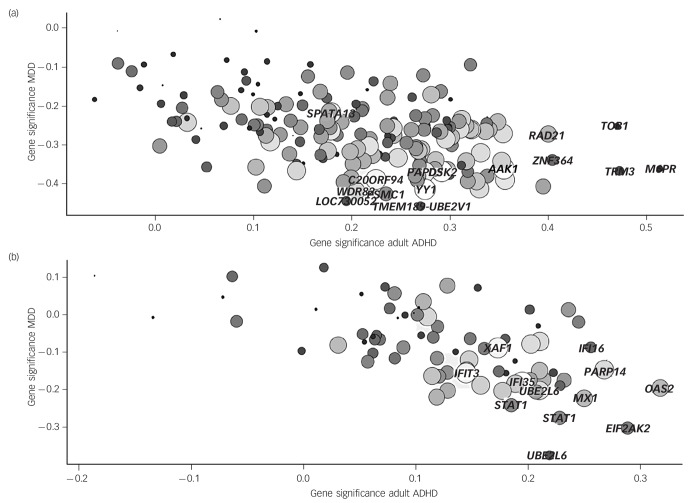
Gene significance for adult attention-deficit hyperactivity disorder (ADHD) *v.* that of major depressive disorder (MDD) for the (a) green and (b) red modules. Size and hue of the circles indicate gene module membership (connectivity). Genes showing highest gene significance are named.

The green (186 probes) and red module (80 probes) eigenvalue estimates per individual correlated negatively with MDD and positively with adult ADHD status (Table 2, Fig. 1). Even though the MDD sample is female-dominated and the adult ADHD sample male-dominated, gender was included as a covariate and therefore does not fully account for this effect. Results also remain significant after additional correction for age. Of the 93 individuals with ADHD, 7 had comorbid MDD. However, these individuals still resembled the pattern of the adult ADHD group more than the MDD group (online Fig. DS5). Wikipathways enrichment analyses through WebGestalt reveal significant enrichment for regulation of toll-like receptor signalling pathway (eight genes, adjusted *P* = 0.02) in the green module. The red module, however, is enriched for immune-related pathways: type II interferon signalling (nine genes, adjusted *P* = 1.8 × 10^−6^), type III interferon signalling (three genes, adjusted *P* = 4 × 10^−3^).

Connectivity mapping (http://apps.lincscloud.org)^19^ revealed 200 compounds corresponding to upregulation of genes in the green module, and 3273 to upregulation in the red module. This implies that application of these compounds to cell lines results in a similar gene expression profile as we find for adult ADHD, and opposite to that for MDD. Among the compounds, there were several tricyclic (amitriptyline, desipramine, nortriptyline, protriptyline, trimipramine) and other (nefazodone, trazodone) antidepressants. In addition, there are steroid-related drugs (among others: betamethasone, cucurbitacin-I, sarmentogenin, wortmannin, alcomethasone, alfadolone, altrenogest, androstanol, beclamethasone, cortisone, corticosterone) and other inflammatory drugs (celecoxib, diclofenac, prostraglandin). Full connectivity mapping results are given in online Table DS2.

Figure 1 depicts gene significance (correlation between phenotype and module eigengene) for MDD and adult ADHD of the red and green module content. Size and colour indicate module membership, our definition of connectivity. Gene significance is highly correlated with gene connectivity in the green (MDD: *r* = −0.45, *P* = 1.7 × 10^−10^, adult ADHD: *r* = 0.36, *P* = 6.2 × 10^−7^) and red (MDD: *r* = −0.43, *P* = 5.9 × 10^−5^, adult ADHD: *r* = 0.45, *P* = 2.9 × 10^−5^) modules, indicating that highly connected hub genes generally show the strongest associations with the phenotypes. The six main green hub genes are *YY1, AAK1, PAK2, C20ORF94* (now *SLX4IP*), *PAPD5* and *SPATA13*. The expression of transcription factor *YY1* indeed shows one of the highest negative correlations with MDD status, together with *TMEM189–UBE2V1, PSMC1, WDR82* and *LOC730052* (now *UBE2V1P2*). *M6PR, TPM3, TOB1, ZNF364* (now *RNF115*) and *RAD21* show highest positive correlation with adult ADHD. For the red module, the hub genes are the interferon genes *IFIT3, IFI35* and *XAF1, PARP14* and two probes for *UBE2L6*. Of these, *PARP14* is highly inversely correlated to MDD and adult ADHD, as are *MX1* and *EIF2AK2*. In addition, two probes for *STAT1* show high gene significance for MDD and *OAS2* for adult ADHD.

### Turquoise module represents overlapping signatures between psychiatric disorders

The turquoise module (2077 probes) is positively correlated with both MDD and childhood ADHD status (Table 2). This effect does not remain significant for childhood ADHD after correction for age. Enrichment analyses reveal significant categories to be cytoplasmic ribosomal proteins (69 genes, adjusted *P* = 2.2 × 10^−22^), electron transport chain (45 genes, adjusted *P* = 10 × 1.6^−8^) and oxidative phosphorylation (25 genes, adjusted *P* = 1 × 10^−3^). The correlation between gene significance and connectivity is *r* = −0.48, *P*<2.2 × 10^−16^ for childhood ADHD and *r* = −0.48, *P*<2.2 × 10^−16^ for MDD. Major hub genes in this module are *ZRANB2, TMEM126B, RPL15, PCNP* and *SRP9*. The genes with highest MDD gene significance are *LOC1001291, ANXA1, RNF13, GMFG* and *H3F3B*. For childhood ADHD these are *SUMO3, RPL22, UTP3, POLE3* and *CCDC50*.

### Disorder-specific signature of the blue module

The blue module (1672) is negatively correlated to childhood ADHD status (Table 2). This effect does not remain significant after correction for age. Enrichment analyses reveal the following significant Wikipathways: integrin-mediated cell adhesion (26 genes, adjusted *P* = 8 × 10^−4^), focal adhesion (35 genes, adjusted *P* = 1 × 10^−3^), prostate cancer (21 genes, adjusted *P* = 6 × 10^−3^) and IL17 signalling pathway (13 genes, adjusted *P* = 0.04). The correlation between childhood ADHD gene significance and connectivity is *r* = −0.5, *P*<2.2 × 10^−16^. Blue hub genes are *WAS, MOBKL2A, C15orf39, G6PD* and *GNAI2*. Genes with highest negative correlation to childhood ADHD disease status are *TMUB2, STAT5B, DENND3, CA4, FCGR3B*.

### Relationship gene expression signatures and previous GWAS findings

We did not find enrichment of GWAS signal or association of the module eigengenes with polygenic risk scores for the modules of interest (online supplements DS2–4 and Figs DS1 and DS2).

## Discussion

### Main findings

This study of whole-blood gene expression of several psychiatric disorders aimed to reveal overlapping gene expression patterns and disorder-specific signatures. The WGCNA on 318 participants revealed seven gene co-expression modules. Of these, two small modules are inversely related to MDD and adult ADHD, the large turquoise module is associated with both MDD and childhood ADHD and finally, the blue module shows a disorder-specific signature for childhood ADHD. No significant results were found for the ASD, ADHD–ASD and control groups. Even though there is some evidence for increased immune-related comorbidities in ASD^20,21^ we do not find an immune gene expression signature for these groups, as we do for MDD and adult ADHD.

### Interpretation and comparison with findings from other studies

Two small modules, red and green, are negatively correlated to MDD status, but positively correlated to adult ADHD status. GWAS on both disorders have not yielded genome-wide significant hits to date.^22,23^ Previous literature about genetic overlap between MDD and ADHD has yielded conflicting results, reporting a genetic correlation between MDD and ADHD,^11^ but no significant overlap in polygenic risk scores.^10^ A reason for these discrepancies could be the lack of distinction between adult ADHD and childhood ADHD in previous studies, which could add to phenotypic heterogeneity.^5^ The group with childhood ADHD may include individuals whose condition will become less severe with age, whereas the adult group with ADHD contains individuals whose condition is chronic. Another reason why we chose to analyse participants with childhood and adult ADHD in separate groups is because of anticipated biological heterogeneity between children and adults in the context of peripheral gene expression. An explanation for our findings would be gender differences in prevalence of MDD and adult ADHD, however, analyses were corrected for gender and stratified analyses yielded the same results (data not shown). We also performed a correlation with the module eigengenes and an indicator of current state of depression (Beck Depression Inventory^24^) within participants, which did not show a significant association (data not shown).

The green module shows enrichment for a cell signalling category and harbours some interesting highly connected hub genes that also show high gene significance, most notably, *YY1, WDR82* and *AAK1. YY1* is a transcription factor involved in many processes including transforming growth factor beta (TGF-β) signalling, but has also shown to be active in histone modification.^25^ In addition *WDR82* (WD repeat domain 82) is part of SET1A/SET1B histone H3K4 methyltransferase complexes,^26^ which genetic pathway analyses has shown to be strongly enriched for association in MDD, bipolar disorder and schizophrenia.^27^ This implication of epigenetic processes provides a mechanism by which environmental influences can exert their influence on gene expression and thereby contribute to the pathology of neuropsychiatric disorders.^28,29^ The green hub gene adaptor-associated kinase 1 (*AAK1*) is of interest because it is a positive regulator of the Notch pathway.^30^ This pathway is traditionally implicated in cell-fate determination during development, but has important function in tissue homeostasis and neuronal plasticity later in life. In addition, it has been implicated to play a role in immune functioning.^31–33^

Even though the immune system has not been implicated in adult ADHD before, there are many studies on its association with MDD although the relationship is still somewhat controversial.^34^ Expression differences in cytokines have been shown to differentiate patients with MDD from ones with bipolar disorder and controls^35^ and studies have shown regulation of the serotonin receptor through cytokines and neurotrophins.^36,37^ Importantly, cytokines and polymorphisms in interleukin genes have been shown to predict antidepressant treatment response.^38,39^ The majority of patients with MDD (70%) and a few (13%) of the participants with adult ADHD in the current study were on antidepressant medication although medication use did not correlate significantly with any gene expression module. However, connectivity mapping revealed that the upregulation of genes in red and green modules as seen in our adult ADHD group coincides with those seen in response to application of a number of tricyclic antidepressants, indicated for the treatment of depression and ADHD with comorbid depression. Results also contained some anti-inflammatory drugs, converging with the module enrichments for immune system genes. This could support the hypothesis that anti-inflammatory drugs such as non-steroidal anti-inflammatory drugs (NSAIDs) might have a role in the treatment of MDD and, our data suggests, also ADHD. Literature on the effects of NSAIDs in patients with MDD is, however, mixed.^40,41^ Our results suggest potential drug repositioning opportunities for NSAIDs for both MDD and ADHD.

Whereas MDD and adult ADHD showed opposite patterns of gene expression changes (the green and red modules), we found an overlapping gene expression signature between MDD and childhood ADHD, in the form of the turquoise module. One possible interpretation of this finding is that childhood ADHD increases risk for developing MDD in later life.^42^ In order to fully untangle this, we would need childhood diagnoses of ADHD in the MDD cohort, but this was not available.

Finally, we find a disorder-specific signature of childhood ADHD in the blue module, which is highly enriched for cell signalling genes. There are two hub genes located on the X-chromosome; *G6PD* and *WAS*. Even though we corrected for gender in our analyses, the childhood ADHD group consisted solely of males, which might explain downregulation of X-chromosome genes, although the blue module is not enriched for genes on sex chromosomes. The significance of the association of childhood ADHD with the turquoise and blue modules disappears when adding age as a covariate in the analyses, but in the absence of a healthy child control group, age and childhood ADHD cohort membership are highly correlated. However, the fact that the childhood dual diagnosis ADHD–ASD group does not show a correlation with these modules suggests that age cannot fully explain these results.

We did not find enrichment of GWAS signal or association of the module eigengenes with polygenic risk scores for the modules of interest (online supplements DS2–4 and Figs DS1 and DS2). This could indicate that the differences in gene expression are driven by environmental rather than genetic factors or, perhaps, that polygenic scores are not yet strong enough in disorders such as MDD and ADHD. One possible known environmental influence on gene expression is smoking. Even though we did not have access to smoking behaviour for all participants, a Fisher's exact test of enrichment of smoking-related genes^43^ did not reveal a significant enrichment for the modules of interest (data not shown). Likewise, a sample-handling or collection effect is unlikely because of initial batch correction and the fact that the healthy controls were for the most part from the same project as the participants with MDD and yet do not show an effect for the relevant modules. In addition, the lack of genetic association could also be the result of the initial GWAS results being underpowered to detect variants associated with MDD, ADHD and ASD.

### Directions for further study

Future research could extend this study to include a broader range of psychiatric disorders, such as psychotic disorders (schizophrenia) and anxiety disorders (obsessive–compulsive disorder, generalised anxiety disorder), in order to better understand the genomic correlates of different syndromes. However, our results have several limitations including power to detect effects in relatively small samples and the reliance upon cross-sectional study designs. Our findings in adult ADHD and MDD will require replication and assessment in different study designs to assess potential therapeutic applications. Also, in this study we examined gene expression in blood but it will be important to determine whether the pattern of results holds true for brain tissue. In ASD, for instance, changes in the expression of a number of genes has been reported to be altered in post-mortem studies.^44^

In conclusion, in a study of gene expression in peripheral blood of patients with psychiatric disorders and healthy controls, we identified both cross-disorder and disorder-specific signatures for adult ADHD and MDD. With the caveats discussed above, they suggest new pathways contributing to distinct pathophysiology in psychiatric disorders and shed light on potential shared genomic risk factors.
